# A molecular method to identify species of fine roots and to predict the proportion of a species in mixed samples in subtropical forests

**DOI:** 10.3389/fpls.2015.00313

**Published:** 2015-05-06

**Authors:** Weixian Zeng, Bo Zhou, Pifeng Lei, Yeling Zeng, Yan Liu, Cong Liu, Wenhua Xiang

**Affiliations:** ^1^Faculty of Life Science and Technology, Central South University of Forestry and TechnologyChangsha, China; ^2^National Engineering Laboratory of Applied Technology for Forestry and Ecology in Southern ChinaChangsha, China; ^3^Huitong National Field Station for Scientific Observation and Research of Chinese Fir Plantation Ecosystem in Hunan ProvinceHuitong, China

**Keywords:** belowground species abundance, fine root, DNA-sequence-based method, PCR, subtropical forest, *trnL*(UAA), intron

## Abstract

Understanding of belowground interactions among tree species and the fine root (≤2 mm in diameter) contribution of a species to forest ecosystem production are mostly restricted by experimental difficulties in the quantification of the species composition. The available approaches have various defects. By contrast, DNA-based methods can avoid these drawbacks. Quantitative real-time polymerase chain reaction (PCR) is an advanced molecular technology, but it is difficult to develop specific primer sets. The method of next-generation sequencing has several limitations, such as inaccurate sequencing of homopolymer regions, as well as being time-consuming, and requiring special knowledge for data analysis. This study evaluated the potential of the DNA-sequence-based method to identify tree species and to quantify the relative proportion of each species in mixed fine root samples. We discriminated the species by isolating DNA from individual fine roots and amplifying the plastid *trnL*(UAA; i.e., tRNA-Leu-UAA) intron using the PCR. To estimate relative proportions, we extracted DNA from fine root mixtures. After the plastid *trnL*(UAA) intron amplification and TA-cloning, we sequenced the positive clones from each mixture. Our results indicated that the plastid *trnL*(UAA) intron spacer successfully distinguished tree species of fine roots in subtropical forests. In addition, the DNA-sequence-based approach could reliably estimate the relative proportion of each species in mixed fine root samples. To our knowledge, this is the first time that the DNA-sequence-based method has been used to quantify tree species proportions in mixed fine root samples in Chinese subtropical forests. As the cost of DNA-sequencing declines and DNA-sequence-based methods improve, the molecular method will be more widely used to determine fine root species and abundance.

## Introduction

Fine roots (≤2 mm in diameter) are the primary organ used by plants to acquire soil water and nutrients for growth (Jackson et al., 1997; de Kroon, 2007). They contribute a great deal to ecosystem production and the nutrient cycle in forests (Silver et al., 2005; Børja et al., 2008). It has been reported that belowground biodiversity in most forest ecosystems is higher than the aboveground biodiversity (Pärtel et al., 2012). A large proportion of the plant biomass is stored belowground and primary production occurs in the roots (Enquist and Niklas, 2002; Poorter et al., 2012). Furthermore, fine root turnover contributes considerably to net primary productivity (NPP) in forest ecosystems (Gill and Jackson, 2000). Hence, belowground interactions among different plant species play important roles in forest community structure, dynamics, and ecosystem functions (Turnbull et al., 2007; Jones et al., 2011). However, belowground interactions among species and the contributions of a species to ecosystem function are poorly understood compared with the aboveground component (Pecháčková et al., 1999; Mommer et al., 2008). The foremost challenge to understand belowground functions in forests is the ability to identify the fine root tree species. The color, size, epidermal characteristics, and morphological traits of roots from different species are too similar to distinguish visually (Mommer et al., 2010; Ugawa et al., 2010). In particular, in subtropical areas, natural forests have more diverse tree species whose roots intermingle and intertwine so that the fine roots are more difficult to identify.

To investigate fine root distribution and to determine fine root biomass of a specific species in forests with diverse plant species, we not only need to identify the species of fine roots but also must estimate the proportion of each species in the mixed root samples. Some methods have been developed to identify the different plant roots to the species level. Near-infrared reflectance spectroscopy (NIRS) is one of the most advanced techniques to predict species composition and proportions of plant fine roots in mixed samples (Roumet et al., 2006; Lei and Bauhus, 2010). Another approach is using plant wax alkanes and fatty alcohols as chemical markers (Roumet et al., 2006). The two methods are both based on the differences in species-specific chemical composition to separate the different species (Dawson et al., 2000; Mommer et al., 2011). However, the accuracy of estimating the root biomass proportion of each species in mixtures is limited because the chemical properties of plant tissue could be influenced by many factors, including atmospheric CO_2_ concentration, herbivore attacks or other environmental components (Soussana et al., 2005; Mommer et al., 2011).

Given that the DNA sequences of species do not vary with environmental changes, the DNA-based methods are not constrained for identifying and quantifying fine roots to the species level in mixed samples (Linder et al., 2000). Quantitative real-time polymerase chain reaction (qPCR) is a popular molecular genetic approach that could accurately quantify relative abundance in mixed roots (Mommer et al., 2008). However, it is difficult to develop primer sets for a specific plant species of interest. Next-generation sequencing is another DNA-based method (Hiiesalu et al., 2012). However, inaccurate sequencing of the homopolymer region, the amount of time and the special knowledge for data analysis that are required, and the high cost, prevent most laboratories from using this method (Grada and Weinbrecht, 2013).

In this study, we describe a sequence-based, inexpensive method that does not require new primer development to identify and quantify the tree species composition of fine root mixtures. We hypothesized that: (1) the fine roots of different species could be distinguished by the plastid *trnL*(UAA; i.e., tRNA-Leu-UAA) intergenic intron, because the plastid *trnL* (UAA) intron has very conserved primers and a robust amplification system (Taberlet et al., 2007), and (2) the DNA-sequence-based method might predict the relative proportion of each species in the mixed fine root samples created using different fresh weight ratios. This study included two novel features. First, our study examined subtropical tree species. To our knowledge, this is the first time that a DNA-sequence-based method was used to identify and quantify fine roots in Chinese subtropical forests. Second, all samples were collected directly from wild forest fields rather than from laboratory-planted saplings.

## Materials and Methods

### Leaf and Fine Root Sample Collection

The classical method to identify a tree species is based on the aboveground parts, particularly leaf, and fruit morphological traits. To evaluate the potential of a DNA-sequence-based method for identifying tree species in fine root mixtures, we performed a BLAST search on fine root DNA sequences in NCBI (National Center for Biotechnology Information) according to the differences in DNA fragments. In case the DNA sequence of some tree species in Chinese subtropical forests would not appear in a BLAST search, we collected leaf and fine root samples simultaneously from a given tree species. When the DNA sequences could not be searched using BLAST, we used the DNA sequence of leaf samples as the reference for the species and matched the DNA sequence of the root samples to the leaf reference. If the DNA sequences between leaves and fine roots of a specific tree species matched, the specific tree species is determined. Using this approach, we ascertained the species of the collected fine root samples and discriminated the species from other mixed species or mycorrhizal fungi.

We sampled the leaves and fine roots of 11 tree species from the forests in Dashanchong Provincial Forest Park (28°23′58^′′^–28°24′58^′′^ N, 113°17′46^′′^–113°19′8^′′^ E) in Changsha County, Hunan Province, China. The climax vegetation in the Park is subtropical evergreen broadleaved forest, but due to past disturbance, there are diverse forests (Liu et al., 2013). The 11 tree species selected in this study consisted of three evergreen coniferous species (*Cryptomeria japonica*, *Cunninghamia lanceolata*, and *Pinus massoniana*), three deciduous broadleaved species (*Choerospondias axillaris*, *Liquidambar formosana*, and *Quercus fabri*), and five evergreen broadleaved species (*Adinandra hainanensis*, *Cyclobalanopsis glauca*, *Litsea coreana*, *Loropetalum chinense*, and *Symplocos bogotensis*). We collected leaf samples from 11 tree species visually through morphological identification and obtained fine root samples of the identical species by tracing root systems from the stem base of the tree identified by leaves. The samples were placed in centrifuge tubes (10 ml) marked with numbers and immediately stored in liquid nitrogen. After the samples were transported to the laboratory, the tubes were stored at -70°C for later analysis.

### Genomic DNA Isolation

Using tweezers, we took out fine root samples from the tubes and then cleaned them with tap water to remove soil particles and exclude dead material. The samples were then dried on filter paper. Each sample of the fresh leaves and individual roots was weighed to 0.1 g (fresh weight) and then ground in liquid nitrogen. Each ground sample was collected and placed in a sterile centrifuge tube (1.5 ml). The DNA of each species for the ground sample in the tube was extracted using the Plant DNA Kit (Tiangen Biotech (Beijing) Co., Ltd.), following the manufacturer’s protocol. Then, the DNA was eluted into a sterile centrifuge tube (1.5 ml) by 70 μl of dd H_2_O and stored at -20°C for further study.

### PCR Amplification

To distinguish tree species on the molecular level, we selected the plastid gene *trnL*(UAA) intron sequence. The DNA sequence of each species was amplified using the primers c and d (Taberlet et al., 1991; Fisk et al., 2010) located in conserved intron sequences, which is a primer set that has been shown to distinguish a wide variety of plant species (Brunner et al., 2001). The PCRs were performed on an Eppendorf Mastercycler in a total volume of 20 μl containing 3 μl of 10x buffer (15 mM Mg^2+^ plus, TaKaRa Ltd.), 2 μl of each dNTP (2.5 mM, TaKaRa Ltd.), 1 μl (10 μM) each of the primers, 0.4 μl Taq DNA polymerase (5 U/μl, TaKaRa Ltd.), and 1.5 μl of template DNA with the following profile: 5 min denaturation at 94°C and 29 cycles of 30 s denaturation at 94°C, 30 s annealing at 52°C, 1 min extension at 72°C, followed by a final extension at 72°C for 5 min. The PCR products were detected by electrophoresis through a 1.0% agarose gel in 0.5 × TAE.

### Species Identification of Fine Roots

The PCR products of individual leaf and root samples were sequenced. At first, the sequence information for each species was acquired by sequencing the PCR products of the individual leaf samples. Accordingly, we constructed the plastid *trnL*(UAA) intron sequence database for the leaf samples from 11 tree species. The sequence information for the leaf samples for each tree species was identified to species by BLAST searches of the NCBI database (Altschul et al., 1997; Fisk et al., 2010). Then, we obtained sequence information for the fine root sample for each tree species. To facilitate the analysis of roots samples and identify tree species of fine roots, we aligned the DNA sequence from root samples to our own database established by leaf samples.

### Proportion Prediction for Mixed Root Samples

To investigate the relative proportion of a tree species in the mixed fine roots, we developed a DNA-sequenced-based method. Because the objective of this study was only to test the reliability of this method, we created two types of fine root mixtures; i.e., a two species mixture and a three species mixture. For the two species mixture, we randomly chose two deciduous broadleaved species (*L. formosana* and *C. axillaris*). Fine root samples of *L. formosana* and *C. axillaris* were mixed to form five different proportions of mixtures (1:0, 1:3, 1:1, 3:1, and 0:1 based on the fresh weight). For the three species mixture, we used the fine root samples of *L. formosana, C. axillaris*, and *C. glauca* to form three different proportions of mixtures (1:2:3, 2:3:1, and 3:1:2 based on the fresh weight). Due to the influences of fine root diameter on the DNA quantification (Fisk et al., 2010), we only chose fresh fine roots with diameters less than 2 mm, and then weighed fine root samples and mixed them to form different mixtures. To reduce the deviation, each mixture was replicated four times. There were 20 two species mixtures samples and 12 three species mixtures samples.

Considering the different DNA concentration and integrity extraction efficiency among the different species, we detected the concentration of each extracted DNA from the same weight (0.1 g fresh weight) of individual fine roots of *L. formosana*, *C. axillaris*, and *C. glauca*, respectively, using a BioPhotometer Plus (Eppendorf). Then, each mixed sample was weighed to 0.1 g (fresh weight) and the DNA was obtained following the DNA extraction method described above. We amplified the plastid *trnL*(UAA) intron region by PCR with the same reaction system and processed it as described above. To remove excess primers, buffer, dNTPs, and polymerase after amplification, the PCR products of the mixed roots samples were purified from the gel using a Gel Extraction kit (Tiangen Biotech (Beijing) Co., Ltd.) according to the manufacturer’s instructions. The purified DNA fragments were then cloned into pMD®18-T easy vector (TaKaRa Ltd.) following the manufacturer’s directions. Next, the recombinant plasmid was transformed into DH5α *E. coli* competent cells. Positive clones using 100 mg/L Amp (Ampicillin), 0.1 M IPTG (Isopropyl β-D-1-thiogalactopyranoside) and 40 mg/L X-Gal (5-Bromo-4-chloro-3-indolyl β-D-galactopyranoside) and confirmed by PCR. The PCR reaction system and process also followed the above steps. At last, from each sample of the two species mixture, 20 positive clones were randomly chosen to sequence. From each sample of the three species mixture, 30 positive clones were randomly chosen to sequence.

**FIGURE 1:**
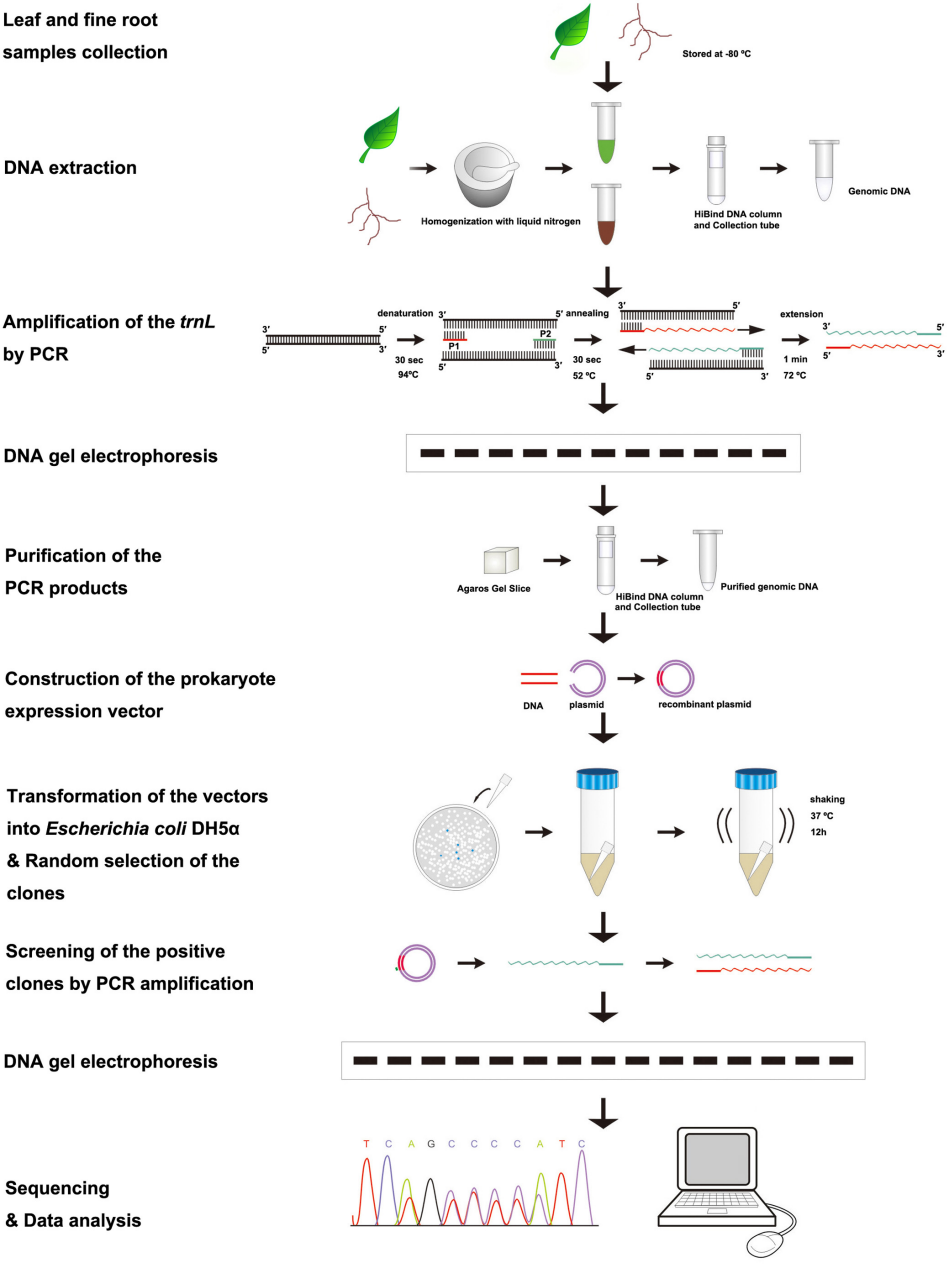
**Flowchart of the procedures to identify tree species and to quantify the proportion of a species in the mixed fine root samples**.

All positive clones were sequenced. At first, we established our own database that contained the plastid *trnL*(UAA) intron sequence of the 11 species, so that each measured sequence could be used in a BLAST search of the established database to confirm the origins of each sequence using GENEDOC and DNAMAN V6 software. Because the sequences of fine roots represent their tree species, one positive clone sequence is used to count the relative proportion of a species. Accordingly, in the two species mixture sample, the ratio of a positive clone sequence represents one-twentieth of the total predicted ratio based on the DNA sequence method. In the three species mixture sample, the ratio of a positive clone sequence accounts for one-thirtieth of the total predicted ratio based on the DNA sequence method. Therefore, the predicted relative proportion based on the DNA sequence method of each species in the fine root mixture samples was estimated by summing up all clone sequence ratios for the species. The relative proportion of a species in each mixture sample was calculated using the Excel 2007 program. The experimental procedures are presented in **Figure 1**.

### Data Analysis

To evaluate the reliability of the DNA-sequence-based method for estimating the relative proportion of a tree species in fine root mixtures, we compared the estimated relative proportion with the actual proportion of fresh fine root weight when mixed. A linear regression was performed to examine the relationship between the actual weight percentage and the predicted percentage based on the DNA sequence method. The regression function, determined efficiency (*r*^2^), and significant degree were obtained for each tree species in the two species fine root mixture and the three species fine root mixture, respectively. Data analysis was performed using the JMP software package (SAS Institute, 1996).

## Results

### Tree Species Identification

**FIGURE 2:**
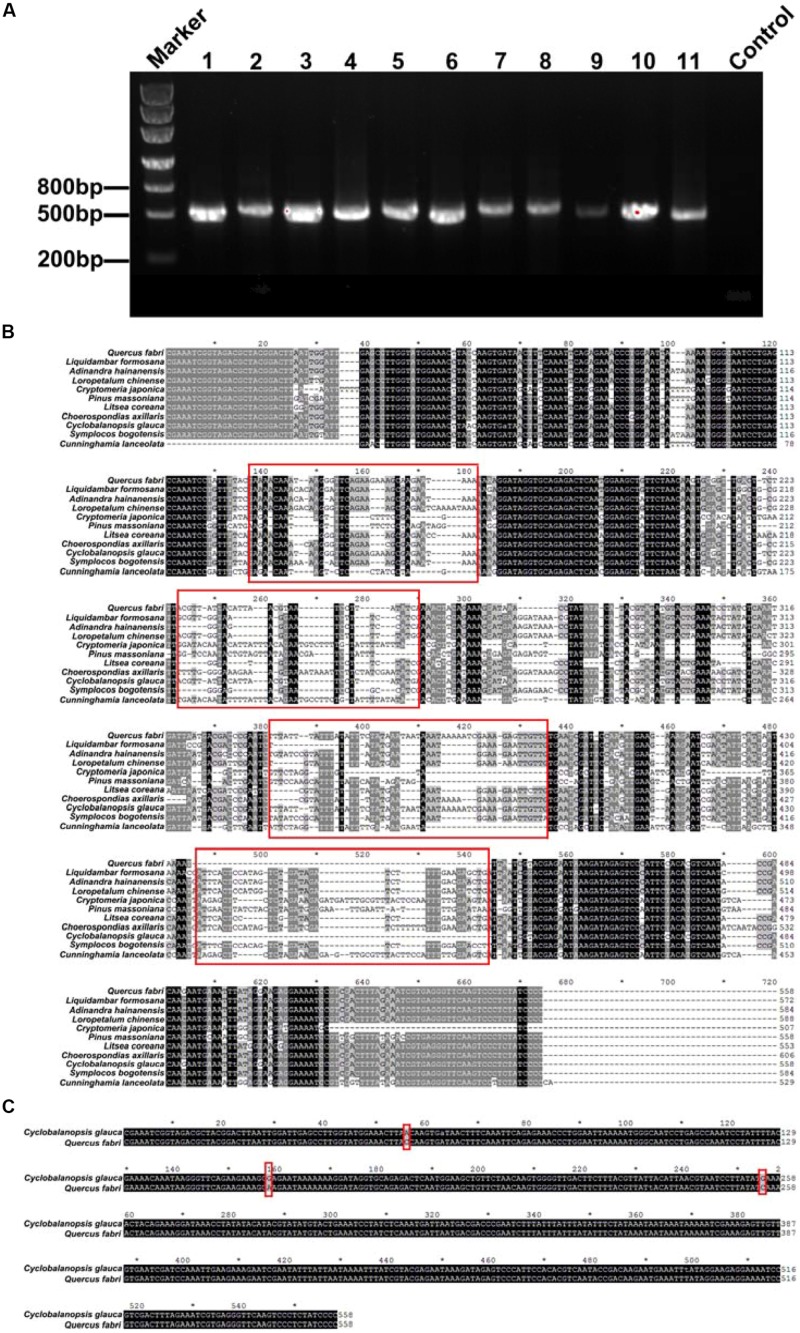
**(A)** Electrophoresis graph of the polymerase chain reaction (PCR)-amplified plastid *trnL*(UAA) intron region. The numbers 1–11 represent different species (1- *Quercus fabri*, 2- *Choerospondias axillaris*, 3- *Symplocos bogotensis*, 4- *Cunninghamia lanceolata*, 5- *Loropetalum chinense*, 6- *Litsea coreana*, 7- *Liquidambar formosana*, 8- *Pinus massoniana*, 9- *Cyclobalanopsis glauca*, 10- *Adinandra hainanensis*, and 11- *Cryptomeria japonica*). **(B)** BLAST results of the plastid *trnL*(UAA) intergenic spacer/intron of the 11 different tree species; the red frames represent significantly different sequence spacers. **(C)** BLAST results of the plastid *trnL*(UAA) intron of *C. glauca* and *Q. fabri*.

The plastid *trnL*(UAA) intergenic spacer/intron of the leaves and roots of 11 plant species were sequenced successfully (**Figure 2A**). The 11 plant species possessed unique fragment lengths. All the sequences of leaf and fine root samples were BLAST searched through NCBI. We found that eight species (*C*. *japonica*, *C*. *lanceolata*, *P*. *massoniana*, *C*. *axillaries*, *L*. *formosana*, *A*. *hainanensis*, *L*. *chinense*, and *S*. *bogotensis*) were consistent with their corresponding species names in NCBI. However, the sequences of the other three species (*C*. *glauca*, *Q*. *fabri*, and *L*. *coreana*) in this study aligned with different species (i.e., *Q. gilva*, *Q. serrata*, and *Cinnamomum insularimontanum*, respectively) in NCBI. Because the plastid *trnL*(UAA) intron sequences between the leaves and fine roots of the same plant species were identical, we are sure that the fine roots of the target species could be discriminated from other species and mycorrhizal fungi. Thus, the plastid *trnL*(UAA) intergenic spacer/intron sequenced in this study could be used to identify the tree species of fine root samples.

In addition, the plastid *trnL*(UAA) intron sequences differed among the tree species (**Figure 2B**). In general, the 11 tree species had enough variance to distinguish plant species, with the exception of *C. glauca* and *Q. fabri*, which have only three different bp (**Figure 2C**). The amplicon sizes ranged from 508 to 606 bp for the 11 species. The longest was *C. axillaris* = 606 bp and the shortest were *C. japonica* = 508 bp; *Q. fabri*, *C. glauca,* and *P. massoniana* = 558 bp; and *S. bogotensis* and *A. hainanensis* = 584 bp (**Figure 2B**; **Table 1**). The range of G+C% of the 11 tree species varied between 33.8 and 39.1% (**Table 1**).

**Table 1:** List of the information for the plastid *trnL*(UAA) intron of the different tree species.

Tree species	NCBI accession no.	The size of the PCR production (bp)	G+C%
*Liquidambar formosana*	KC588388	572	38.3
*Choerospondias axillaris*	AY594544	606	36.3
*Cunninghamia lanceolata*	KC427270	529	35.4
*Quercus fabri*	JN102146	558	33.9
*Cyclobalanopsis glauca*	JN102172	558	33.8
*Adinandra hainanensis*	HQ158574	584	35.5
*Loropetalum chinense*	HM369421	588	35.7
*Cryptomeria japonica*	AP010967	507	34.8
*Pinus massoniana*	KC427272	558	39.1
*Litsea coreana*	JN102156	553	38.2
*Symplocos bogotensis*	AJ430889	584	36.5

### Prediction of Species Proportions in Mixed Fine Root Samples

**FIGURE 3:**
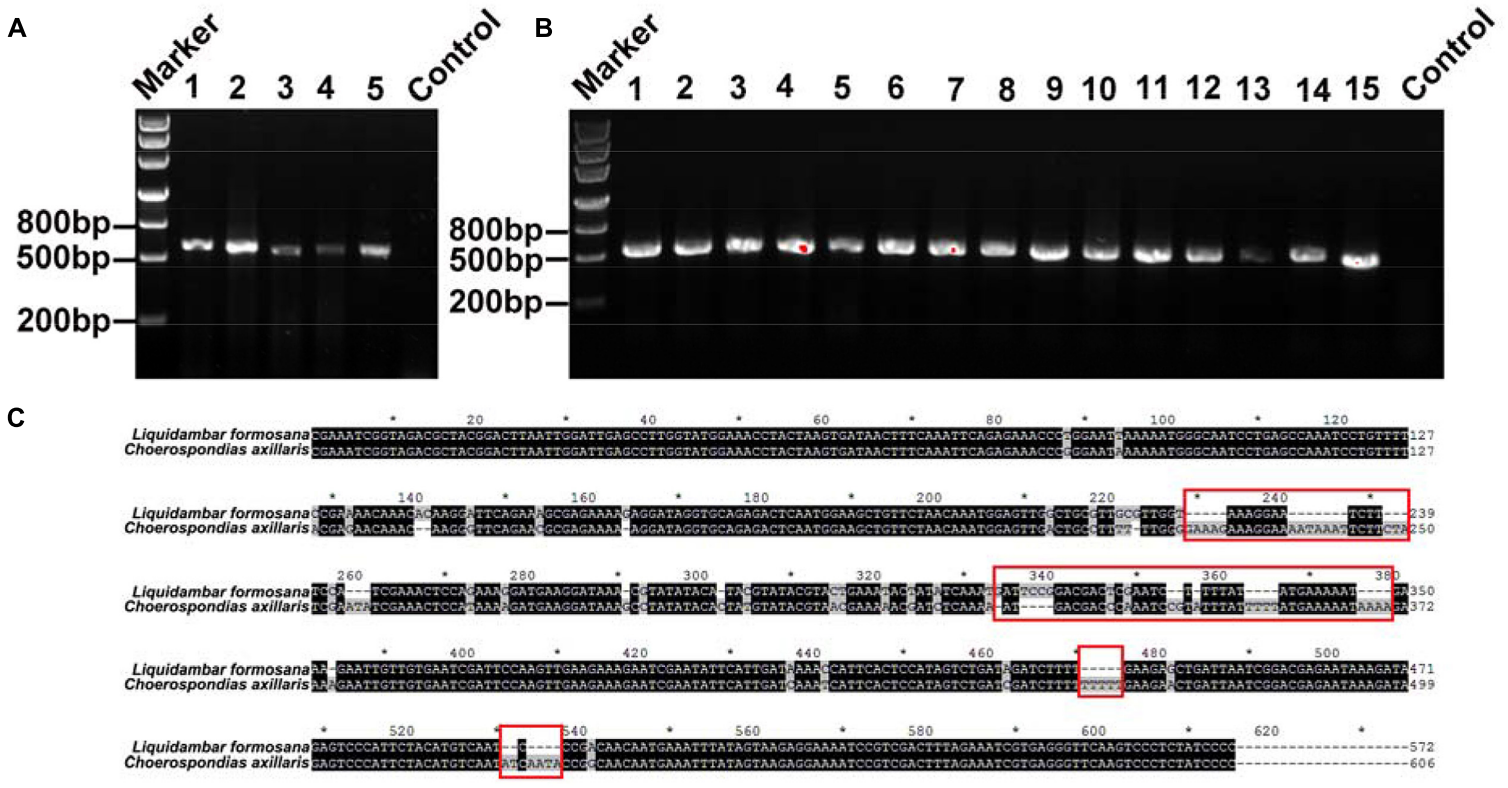
**(A)** DNA gel graph of the PCR products from DNA of mixed samples consisting of two species. The number 1 stands for 0% of *L. formosana* sample, and 2, 3, 4, and 5 represent 25, 50, 75, and 100% of *L. formosana* samples, respectively. **(B)** The numbers 1–15 all denote positive PCR products for the 50% of *L. formosana* samples. **(C)** Blasting the sequences of *L. formosana* and *C. axillaris*; the red frames point to the obvious different sequence spacer electrophoresis picture of positive clone PCRs.

**FIGURE 4:**
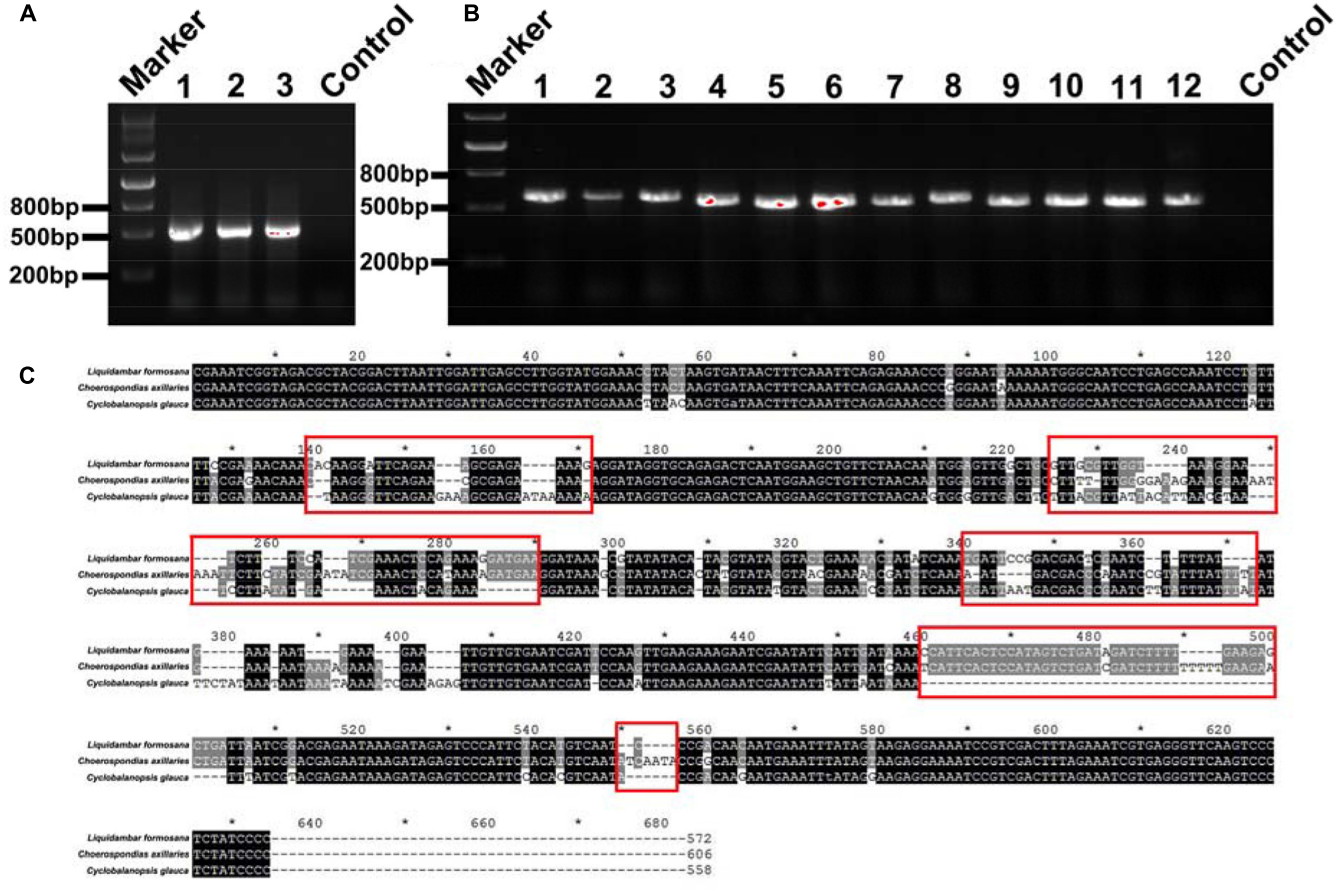
**(A)** DNA gel graph of the PCR products from DNA of mixed samples consisting of three species. The numbers 1, 2, and 3 stand for 1:2:3, 2:3:1, and 3:1:2 mixed ratios of *L. formosana*, *C. axillaris*, and *C. glauc*a samples, respectively. **(B)** The numbers 1–12 all denote positive clone PCR products for the 1:2:3 mixed ratios of *L. formosana*, *C. axillaris*, and *C. glauc*a samples. **(C)** Blasting the sequences of *L. formosana*, *C. axillaris*, and *C. glauca*; the red frames point to the obvious different sequence spacer electrophoresis picture of positive clone PCRs.

**FIGURE 5:**
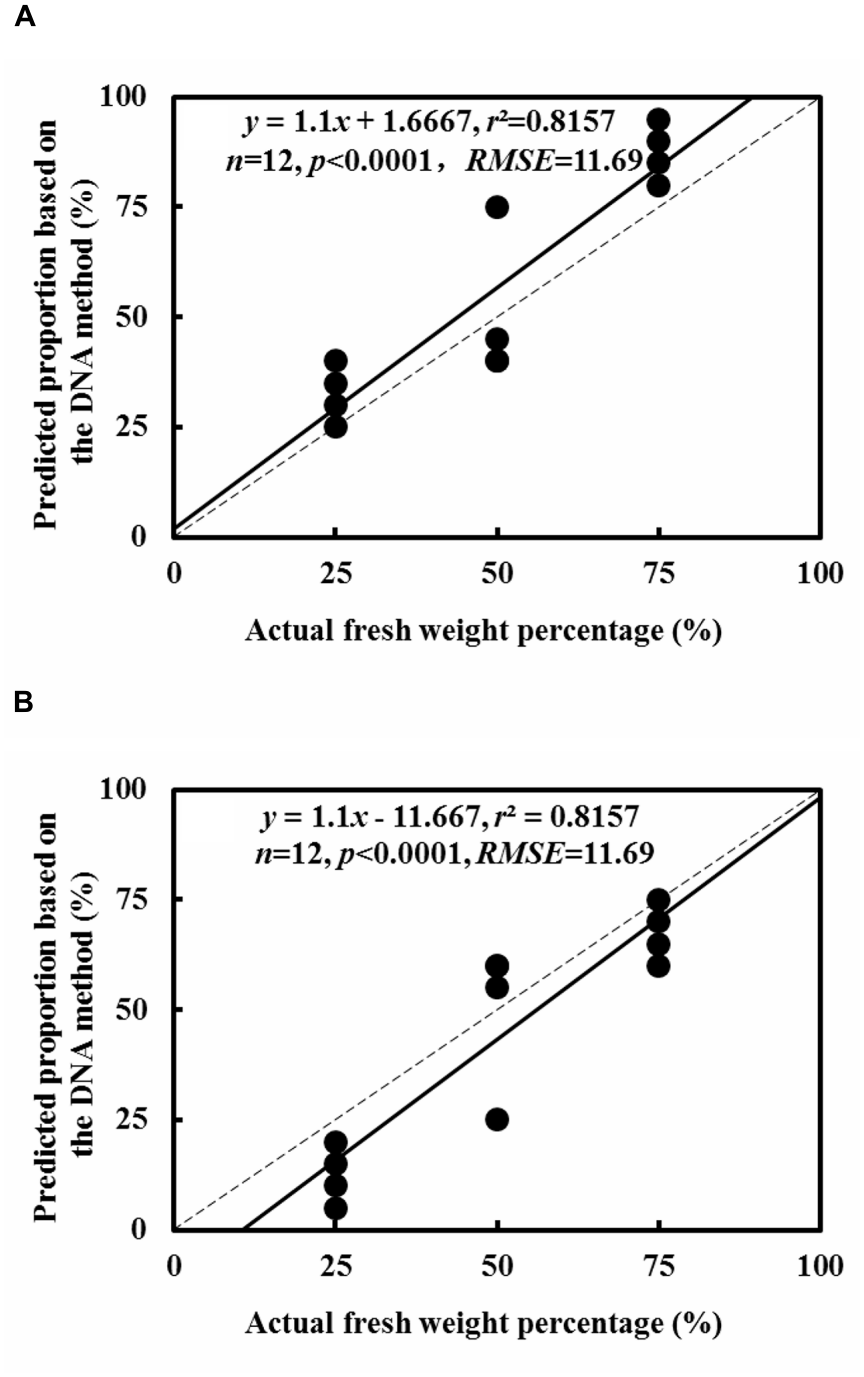
**Comparison of the predicted relative proportion based on the DNA method with the actual relative proportion of fresh weight for *L. formosana***(A)** and *C. axillaris***(B)** fine roots in mixed samples of the two species.** The dash line represents one to one line and the solid line represents the regression line.

In every clone library of replicate mixtures of both, two species and three species, species identity in mixed fine root samples were successfully identified and no other species were detected (**Figures 3** and **4**). For the fine root mixtures of two species, we found a good fitness between the predicted relative proportion based on the DNA sequence method and the actual fresh weight proportion in the mixed samples (*r*^2^ = 0.8157, *p* < 0.0001 for *L. formosana* and *r*^2^ = 0.8157, *p* < 0.0001 for *C. axillaris*; **Figure 5**). Both *L. formosana* and *C. axillaries* had a low and the same value (11.69) of root mean square error (RMSE) for the regressions (**Figure 5**). In the samples of 0% fresh weight of *L. formosana*, *L. formosana* was not detected, whereas in the samples of 100% fresh weight of *L. formosana*, only *L. formosana* was detected. The fresh weight of 1:1 (*L. formosana*: *C. axillaris*) in a sample resulted in an average estimate of 50% *L. formosana* (**Figure 5A**). As the percentage of actual fresh weight of *L. formosana* increased from 25 to 75%, the average predicted proportion also increased, from 32.5 to 87.5% (**Figure 5A**). A similar pattern was found for *C. axillaris* in different mixed proportions (**Figure 5B**).

**FIGURE 6:**
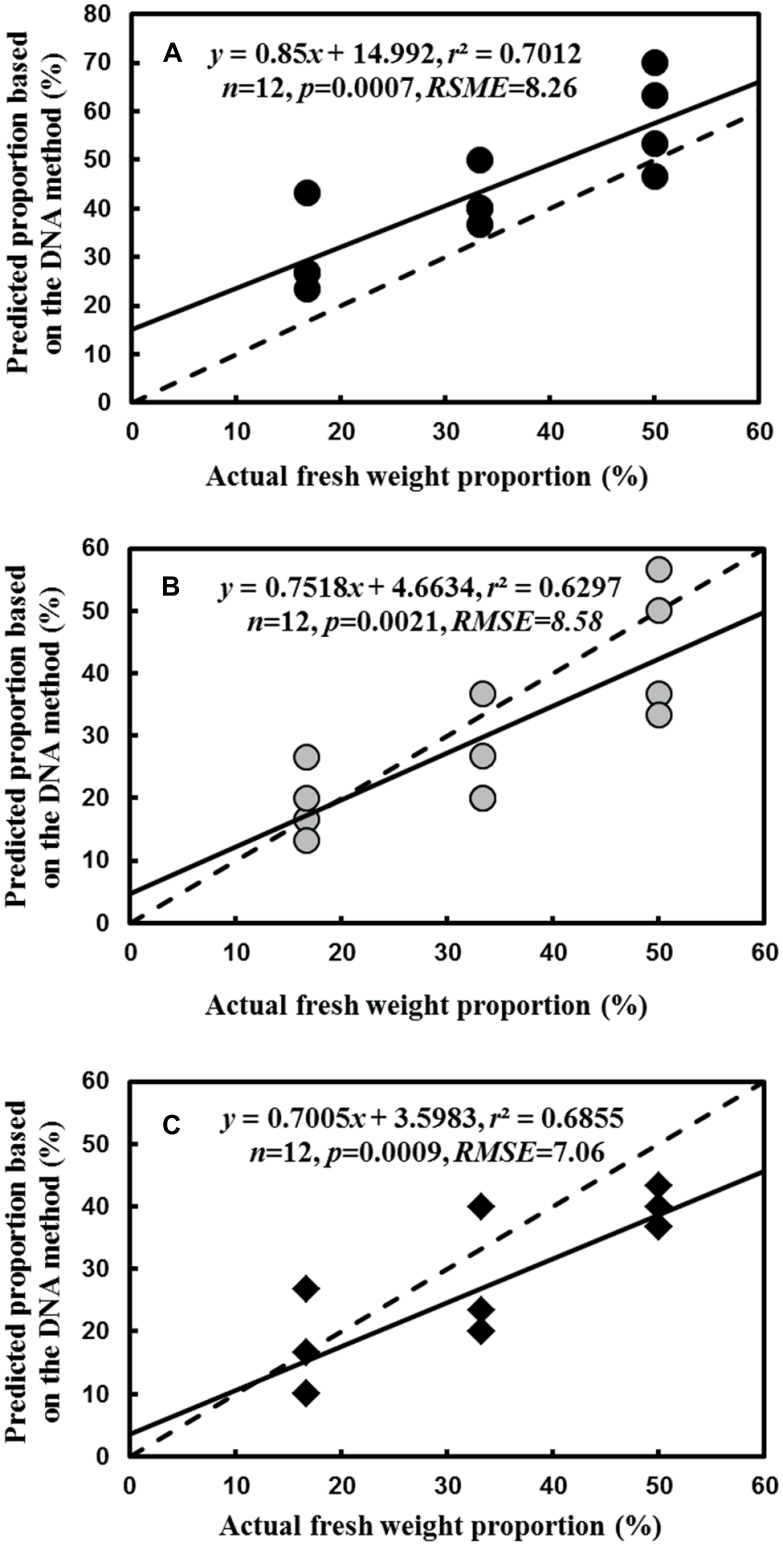
**Comparison of the predicted relative proportion based on the DNA method with the actual relative proportion of fresh weight for *L. formosana***(A)**, *C. axillaris***(B)**, and *C. glauca***(C)** fine roots in mixed samples of the three species.** The dash line represents one to one line and the solid line represents the regression line.

For the fine root mixtures of three species, the predicted relative proportions of each species exhibited a reasonable relationship with the actual proportion of fresh weight in the mixed samples (*r*^2^ = 0.7012, *p* = 0.0007 for *L. formosana*, *r*^2^ = 0.6297, *p* = 0.0021 for *C. axillaries* and *r*^2^ = 0.6855, *p* = 0.0009 for *C. glauca*; **Figure 6**). The values of RMSE for the regressions were very low (8.26 for *L. formosana*, 8.58 for *C. axillaries*, and 7.06 for *C. glauca*; **Figure 6**). The predicted relative proportion based on the DNA sequence method was slightly higher for *L. formosana,* but was slightly lower for *C. axillaris* and *C. glauca*, compared with the actual proportion of fresh weight in the fine root mixture (**Figure 6**). Specifically, when the actual proportions of fresh weight for *L. formosana* were 16.7, 33.3, and 50.0%, the average predicted relative proportions were 30.0, 41.7, and 58.3%, respectively, approximating an average 10.0% overestimation (**Figure 6A**). By contrast, the predicted relative proportions of *C. axillaris* and *C. glauca* were underestimated, with an average prediction of 19.2, 25.9, and 44.2% for *C. axillaries* (**Figure 6B**) and 15.9, 25.8, and 39.1% for *C. glauca* (**Figure 6C**).

## Discussion

### Tree Species Identification

The results of this study indicated that the plastid *trnL*(UAA) intron could be used to identify the 11 tree species of fine roots. We successfully extracted the DNA from the leaf and fine roots of each tree species. Moreover, the plastid *trnL*(UAA) intron sequences of the leaves and roots were fully aligned for a specific species. Based on the database of DNA sequences of leaf samples for each species, we can confirm with confidence that the DNA sequence of the fine root samples belonged to the species identified by the leaf samples, even though the names of *C*. *glauca*, *Q*. *fabri*, and *L*. *coreana* corresponded to different names (i.e., *Q. gilva*, *Q. serrata*, and *C. insularimontanum*) in NCBI. These results support the conclusion of (Hiiesalu et al., 2012) that the plastid *trnL*(UAA) intergenic intron is a powerful DNA plant barcode and that it is possible to use it to identify a large number of different species in the forests. We will submit the three fragment sequences to the NCBI and state that the fragment sequences exist in *C*. *glauca*, *Q*. *fabri*, and *L*. *coreana*.

### Application of the DNA-Sequence-Based Method in Species Composition Quantification

In addition to tree species identification, we also tested whether the plastid *trnL*(UAA) intron sequence could be used to estimate the relative proportion of each tree species in mixed fine roots in a Chinese subtropical forest. Previous studies primarily focused on species identification (Taberlet et al., 2007). There are few studies quantifying root species proportions. Thus, estimating relative species abundance in mixed samples is a new direction (Linder et al., 2000; Mommer et al., 2008). In this study, we developed a molecular method to predict the relative proportion of a species in fine root mixture samples and to examine the reliability of the prediction by comparing the prediction based on the DNA sequence method with the actual fresh weight percentage for each species in fine root mixtures.

Our results showed that the predicted proportions were close to the actual fresh weight proportions in mixed fine root samples consisting of two species; the deviation was within the range of approximately 3–5% (**Figure 5**). This implies that the DNA-sequence-based method was reliable to predict the relative proportion of the fine root mixtures consisting of two species in subtropical forests. This was consistent with the report by Fisk et al. (2010), who applied the DNA-sequence-based approach to quantify the species composition of fine roots. In the study of Fisk et al. (2010), the relative abundance was sensitive to root diameter. Hence, the experiment using fresh fine roots (≤2 mm diameter), ignoring the thick roots and the dead roots, showed that the actual weight and the DNA sequence were relative. In addition, Mommer et al. (2008) conducted similar experiments, using a real-time PCR (qPCR) technique and root samples collected from artificial plant communities that were grown for two growing seasons prior to the test. By contrast, our experimental materials were directly collected from the natural forest field. In the wild, there are many unknown factors that influence the results. Our results agree well with the reports of Mommer et al. (2008) and have significant reference value to study the natural forest field. In the study of Mommer et al. (2008), only four species were reported because the primer was not specific to other plant species. Moreover, we investigated the proportion of woody species, but Mommer et al. (2008) tested herbal species.

However, for the fine root mixture consisting of three species, the accuracy of the predicted relative proportion for each species decreased, with a maximum 15% overestimation for *L. formosana* and 20% underestimation for *C. axillaris* and *C. glauca*. The deviation in the present study was higher than reported by Mommer et al. (2008) but was lower than reported by Fisk et al. (2010). The high deviation in this study could be attributed to different DNA concentrations in the fresh fine root of each tree species. For example, our study found that the extraction concentration of *L. formosana* (∼3370 ng.μl^-1^) was modestly higher than *C. axillaris* (∼3160 ng.μl^-1^) and *C. glauca* (∼2970 ng.μl^-1^). To reduce the deviation, the differences of DNA concentration in fresh fine root among different species could be taken into account for modifying the prediction regression. In summary, our approach has much practicability and generalization. This method can not only distinguish a large number of species but also reliably estimate the relative proportion of individual species. In the future, we will test a real-time PCR (qPCR) technique based on the plastid *trnL*(UAA) intron to design the primers.

### Consideration of Factors to Improve the Accuracy of Species Composition Quantification

These results present some variability. We estimated several potential sources of bias that could affect the quantification of the relative abundance of species. Here, we suggest some solutions. First, the efficiencies of DNA extraction were different among species, because the tissue chemistry and the root diameter might influence the DNA extraction (Guo et al., 2004; Fisk et al., 2010). Second, differential desiccation of monoculture samples would lead to weighing errors and consequently cause enormous bias (Mommer et al., 2008). Therefore, we should select roots of a similar degree of dryness as the specimen material. Thirdly, the difference in gene copy number, G+C%, and the size would all affect the efficiency of amplification (Wintzingerode et al., 1997), and may mean that the amplification cycle number could be lower (Suzuki and Giovannoni, 1996). As molecular experiments, all the DNA-based methods might face these same problems. However, the high reproducibility showed that the deviation was unlikely to result from the PCR process (Mommer et al., 2008). Finally, screening the positive clones in this study was random, so we need to choose more clones and include more reduplicates to lower the deviation and to improve the accuracy of our results. Although there are some biases, the results show that this method is reliable and practicable. In the future, it should be determined whether this result is consistent in mixed samples containing more species. More research remains to estimate whether this approach can be applied to natural conditions other than subtropical forests.

Overall, we have shown that the amplification of the plastid *trnL*(UAA) intergenic intron can distinguish tree species of fine roots in subtropical forests, and we present a new DNA-based method to quantify species composition in mixed fine root samples collected in nature. These findings are an important forward step to the future in belowground research, such as plant interactions or specific species contributions to the ecosystem.

### Conclusion

Our results showed that the amplification of the plastid *trnL*(UAA) intergenic intron successfully distinguished the 11 subtropical tree species of fine roots and a method could be developed to predict fine root species composition in mixture communities. This study provides an example of the DNA-sequence-based method for the study of belowground interaction and functions in forests. It is the first time that the molecular method has been used to quantify tree species composition in mixed fine roots in Chinese subtropical forests. The DNA-based approach could be widely used to reveal belowground processes.

## Author Contributions

Idea and study design: WX and BZ; data collection and analysis: WZ, YL, and CL with the support of BZ; writing of the manuscript: WZ, WX, BZ, PL, and YZ.

## Conflict of Interest Statement

The authors declare that the research was conducted in the absence of any commercial or financial relationships that could be construed as a potential conflict of interest.
